# Severe malaria readmissions in Northern Uganda: a cross-sectional study

**DOI:** 10.1186/s12936-025-05307-8

**Published:** 2025-02-27

**Authors:** James Olum, David Mukunya, Brendah Nambozo, Ritah Nantale, Faith Oguttu, Joshua Epuitai, Ivan Lume, Benon Wanume, Peter Olupot-Olupot, Daphine Amanya, Abel Kakuru

**Affiliations:** 1 Department of Community and Public Health, Faculty of Health Sciences, Busitema University, PO Box 236, Tororo, Uganda; 2 Department of Peadiatrics, Mbale Regional Referral Hospital, Mbale, Uganda; 3 Department of Nursing, Faculty of Health Sciences, Busitema University, PO Box 236, Tororo, Uganda; 4 Department of Internal Medicine, Faculty of Health Sciences, Busitema University, PO Box 236, Tororo, Uganda; 5 Mbale Clinical Research Institute, Pallisa Road, PO Box 291, Mbale, Uganda

**Keywords:** Children under 5 years of age, Readmission, Associated factors, Severe malaria, Children, Uganda

## Abstract

**Background:**

Malaria is a critical global health issue, particularly for children in endemic regions. However, factors associated with recurrent severe malaria in children under 5 years of age in Northern Uganda are poorly understood. This study aimed to identify factors associated with readmission due to severe malaria within six months post-discharge among children in this age group.

**Methods:**

A cross-sectional study was conducted in Otuke district, encompassing twelve health facilities. A total of 760 caregivers of children admitted with severe malaria were interviewed, and hospital records were reviewed to verify the readmission data. The primary outcome assessed was readmission with severe malaria within six months after initial discharge. Data analysis was performed via Stata version 15.0.

**Results:**

The prevalence of readmission with severe malaria among children under 5 years of age was 26.8% (198/739). Factors significantly associated with readmission included having sickle cell anaemia [adjusted prevalence ratio (aPR) 1.72; 95% confidence interval (CI) (1.95–3.14)], living in houses constructed with straw and thatch walls [(aPR 2.10; 95% CI (1.19–3.69)] and seeking care after 12 h when the child has a fever [aPR 2.01; 95% CI (1.23–3.29)].

**Conclusion:**

The findings indicate a high proportion of severe malaria readmissions in children under 5 years of age. Sickle cell anaemia, living in houses built using straw and thatch walls and seeking care after 12 h when a child has fever were the key risk factors for readmission with severe malaria. This study highlights the importance of targeted post-discharge interventions, such as prophylactic anti-malarials in addition to bed nets, to prevent recurrent infections especially among children with sickle cell disease. In addition, improvements in housing quality and timely treatment of children with malaria are essential for reducing the burden of malaria, particularly in endemic regions.

## Background

There were 249 million malaria cases worldwide in 2022 [1]. Sub-Saharan Africa (SSA) has the highest burden, accounting for 94% of the global burden of malaria cases [1]. Severe malaria is a life-threatening condition caused by infection with *Plasmodium* parasites, most commonly *Plasmodium falciparum* [2]. It occurs when malaria progresses from an uncomplicated state to one involving serious complications, often due to delayed treatment or the host’s inability to control the infection [3]. Severe malaria is characterized by clinical or laboratory signs of vital organ dysfunction or metabolic abnormalities. According to the World Health Organization (WHO) [3], severe malaria is defined in anyone or a combination of the following clinical or laboratory criteria: severe anaemia (Hb less than 5 g/dl), prostration (generalized weakness so that the person is unable to sit, stand or walk without assistance), and shock (compensated shock with capillary refill time ≥ 3 s or a temperature gradient and no hypotension). Decompensation shock is defined as a systolic blood pressure < 70 mmHg in children with evidence of impaired perfusion (cool peripheries or prolonged capillary refill), spontaneous haemorrhage (recurrent or prolonged bleeding from nose gums or venipuncture sites; haematemesis or melaena), multiple convulsions (2 or more convulsions in 24 h), impaired consciousness (Blantyre coma score < 3), jaundice [plasma bilirubin > 50 µmol/L (> 3 mg/dl)], pulmonary oedema/respiratory distress (Kussmaul’s breathing manifesting as deep breathing with signs of increased work of breathing) and (haemoglobinuria), hyper-parasitaemia (greater than 10% in highly endemic areas), acidosis (venous plasma lactate > 5 mmol/L), hypoglycaemia (< 2.2 mmol/L or < 40 mg/dL), lactic acidosis (lactate > 5mol/L), and renal failure (serum creatinine > 3 mg/dL) [3]. Prompt recognition and treatment of severe malaria are critical, as they can lead to multiorgan failure or death, especially in young children and pregnant women.

In 2022, an estimated 608000 deaths occurred globally due to malaria, more than 50% of which were in SSA [1]. Approximately seventy-six percent of these deaths occur in children under 5 years of age [1]. Uganda accounts for five percent of the malaria cases in SSA [4]. This disease is one of the leading causes of recurrent hospitalization among children in Uganda [5]. A recent systematic review revealed that there is up to 33% risk of all-cause mortality among children and/or being readmitted within the first six months after discharge [6]. Moreover, readmission due to severe malaria has negative implications for parents and health systems, such as increased financial burden and prolonged hospitalization [7].

The Lango subregion in Northern Uganda is among the areas in the country where malaria is endemic, with high disease prevalence rates of 23% [8]. The government has instituted strategies to reduce malaria transmission, such as indoor residual spraying and the distribution of insecticide-treated mosquito nets [9–11]. Despite these strategies, the incidence of malaria remains high, especially in children under 5 years of age. Understanding the burden and factors associated with the readmission of children with severe malaria could contribute to the design of new or modified existing interventions for the prevention and control of malaria transmission in highly endemic areas. In addition, there is limited information on the burden and factors associated with the readmission of children with severe malaria in Northern Uganda. This study assessed the factors associated with the readmission of children under 5 years of age with severe malaria within six months in Northern Uganda.

## Methods

### Study design, setting, and population

**Fig. 1:**
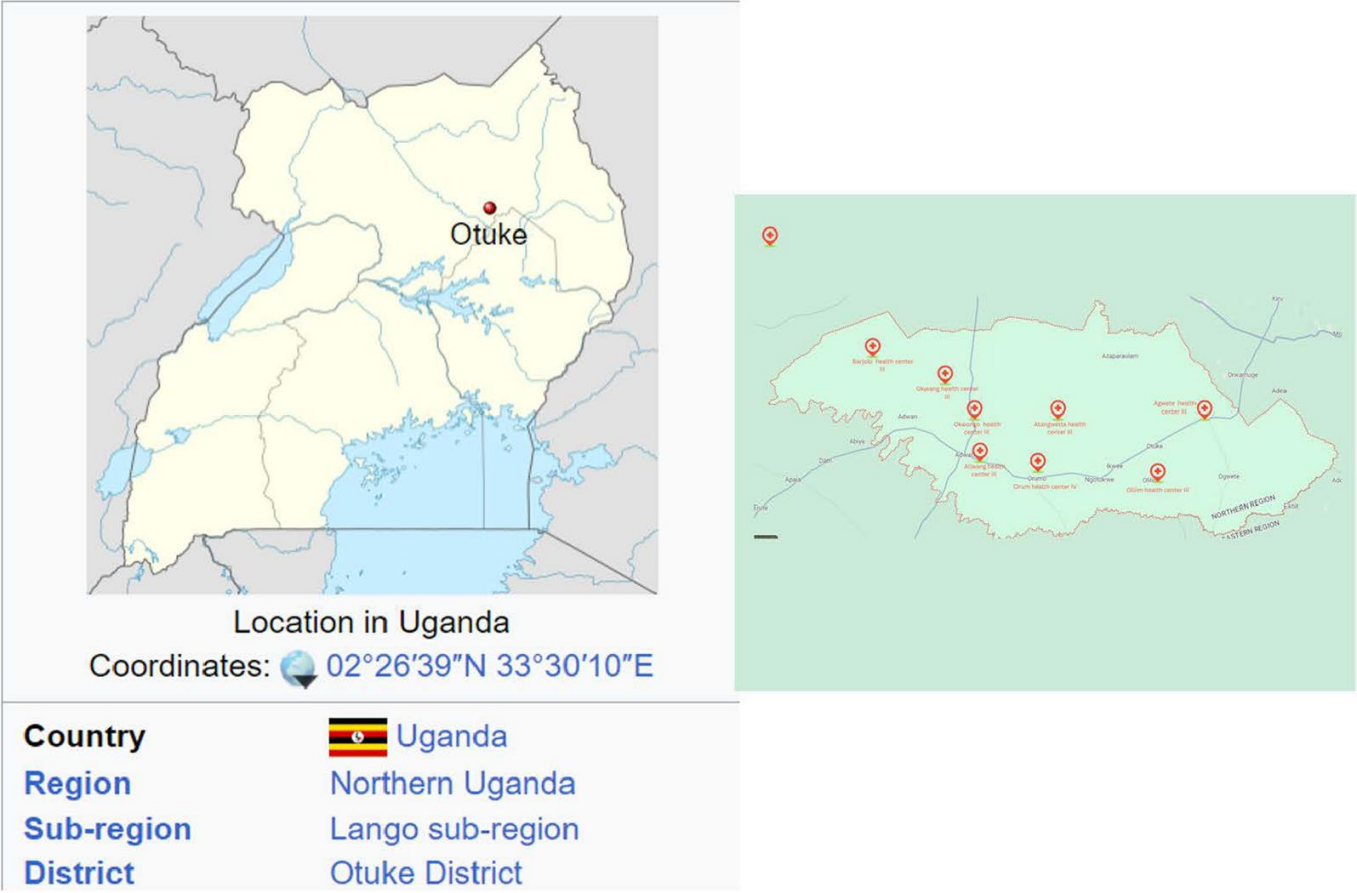
Map of Uganda showing health facilities in Otuke district where the study was conducted

This was a cross-sectional study among children under 5 years of age admitted with severe malaria between March 2023 and May 2023 in Otuke district in Northern Uganda. This study was conducted within nine public health facilities and three private clinics. These included Orum Health Centre IV and eight Health Centre IIIs (Okwongo, Okwang, Barjobi, Aliwang, Atangwatta, Olilim, Ogwette, Christina). Malaria is endemic in this area, and many cases are registered annually (DHIS from the Otuke district). Figure 1 shows the study area and sites.

This study included children under 5 years of age admitted with severe malaria at the selected health facilities. Written informed consent was obtained from all caregivers of the children before data collection. Children were excluded from the study if they did not have an adult or emancipated minor as a caregiver.

### Data collection

Trained research assistants, including nurses and clinical officers, collected data via an electronic questionnaire via Kobotool box software. The patient registers were checked every day, and a list of children admitted with severe malaria was generated. The caregivers of the selected children were approached, screened, and written informed consent was obtained before enrollment in the study. Thereafter, the questionnaire was administered to the caregivers. For children who had been readmitted, the research assistant further checked their medical records or the patient registers to confirm the readmission status.

The primary outcome variable was readmission with severe malaria. Severe malaria diagnosis was defined on the basis of the WHO criteria [3], which have been used in similar settings in Uganda [12]. Parents and caretakers of children who met this case definition were interviewed. The exposure variables studied included the age of the child, sex of the child, maternal age, maternal education, paternal education, socioeconomic status of the parents/caregiver, comorbidity (sickle cell disease and HIV), knowledge of malaria transmission, wall materials of the house, use of insecticides, use of mosquito nets, clearing bushes, draining stagnant water, and time to and seeking treatment when a child has a fever, among others. Health facility registers, discharge forms, and patients’ clinical notes were checked to confirm readmission cases and first-time admissions.

### Data analysis

The data were analysed via Stata version 15.0 (Stata Corp LLC, College Station, Texas, USA). Continuous variables were summarized using means with standard deviations and categorical variables using frequencies and percentages. A generalized linear model was used to assess the relationships between the exposure variables and outcome. Factors with a P-value less than 0.2 in the bivariable analysis and those known to be associated with severe malaria admission were added to the multivariable model. Prevalence rates at 95% confidence intervals were calculated. Significance was set at a P-value less than 0.05. For knowledge of the transmission of malaria, each correct answer of the eight questions was given one point. A participant was considered to have good knowledge if their total score was above the mean score of 5.2 ± 2.5 (a score of 0–5.2 was categorized as poor knowledge and a score of 5.3 or more was considered good knowledge).

## Results

### Characteristics of study participants

Seven hundred sixty participants were enrolled and only twenty-one were excluded during the analysis because they had missing records for the previous admissions. Data from 739 participants were analysed. The mean age of the children was 2.3 years (standard deviation ± 1.1). The mean maternal age was 27.8 years (standard deviation ± 6.4), and the mean paternal age was 33.8 years (standard deviation ± 7.7). Only twenty percent (148/739) of the participants were in the poorest wealth quintiles. Only two of the 198 children who were readmitted received post-discharge malaria chemoprophylaxis with dihydroartemisinin-piperaquine. Among the 739 children, 161 had chronic illnesses, including sickle cell disease 41.6 (67/161) and HIV 18 (29/161) (Table 1).

**Table 1:** Characteristics of children admitted with severe malaria in Northern Uganda

Characteristic	severe malaria readmission		Total	P value
	No, n (%) n = 541	Yes, n (%) n = 198	n = 739	
Age of the child, Mean ± SD = 2.3 ± 1.1				
1 year	164 (30.3)	63 (31.8)	227 (30.7)	0.918
2 to 3 years	262 (48.4)	93 (47)	355 (48)	
4 years	115 (21.3)	42 (21.2)	157 (21.2)	
Sex of the child				
Male	279 (51.6)	99 (50)	378 (51.2)	0.705
Female	262 (48.4)	99 (50)	361 (48.8)	
Maternal age (years), Mean ± SD = 27.8 ± 6.4				
< 20	36 (6.7)	11 (5.6)	47 (6.4)	0.639
20 to 24	150 (27.7)	56 (28.3)	206 (27.9)	
25 to 34	249 (46)	99 (50)	348 (47.1)	
≥ 35	106 (19.6)	32 (16.2)	138 (18.7)	
Maternal education				
None/primary	405 (74.9)	156 (78.8)	561 (75.9)	0.537
Secondary	102 (18.8)	32 (16.2)	134 (18.1)	
Tertiary	34 (6.3)	10 (5.1)	44 (5.9)	
Paternal age (years), Mean ± SD = 33.8 ± 7.7				
17 to 24	41 (7.8)	17 (8.7)	58 (8)	0.249
25 to 34	262 (49.5)	103 (52.6)	365 (50.3)	
≥ 35	226 (42.7)	76 (38.8)	302 (41.7)	
Post discharge malaria chemoprophylaxis with Dihydroartemisinin-piperaquine	0 (0)	2 (1)	2 (1.0)	
Wealth indices				
Poorest	86 (15.9)	62 (31.3)	148 (20.0)	< 0.001
Poorer	104 (19.2)	45 (22.7)	149 (20.2)	
Middle	118 (21.8)	34 (17.2)	152 (20.6)	
Richer	124 (22.9)	26 (13.1)	150 (20.3)	
Richest	109 (20.2)	31 (15.7)	140 (18.9)	
Distance from home to the nearest health facility				
≤ 2 km	209 (38.6)	67 (33.8)	276 (37.3)	0.2
3 to 5	213 (39.4)	96 (48.5)	309 (41.8)	
> 5	119 (22)	35 (17.7)	154 (20.8)	
Time to seeking care when the child has a fever				
Within 12 h	394 (72.8)	82 (41.4)	476 (64.4)	< 0.001
Within 13–24 h	61 (11.3)	74 (37.4)	135 (18.3)	
After 24 h	86 (15.9)	42 (21.2)	128 (17.3)	
Do you have a mosquito net				
No	67 (12.4)	22 (11.1)	89 (12)	0.638
Yes	474 (87.6)	176 (88.9)	650 (88)	
Comorbidity (n = 161)				
1. Diabetes	2 (0.7)	1 (1.1)	3 (1.9)	< 0.001
2. Chronic respiratory infection	43 (15.4)	19 (21.8)	62 (38.5)	
3. Sickle cell disease	35 (12.5)	32 (36.8)	67 (41.6)	
4. HIV	26 (9.3)	3 (3.4)	29 (18.0)	

^*^SD: Standard Deviation

### Knowledge of the transmission of malaria and prevention measures used by the participants

The mean knowledge score was 5.2 ± 2.5, and more than half [53.5% (395/739) of the participants had good knowledge. Almost all [98.4% (727/739)] reported the use of mosquito nets, 52.0% (384/739) reported clearing bushes, 28.1% (208/739) reported closing windows and doors, and 49.1% (363/739) reported draining stagnant water (Table 2).

**Table 2:** Knowledge of the transmission of malaria and prevention measures used by caregivers of children under 5 years of age with severe malaria in Northern Uganda

Variable	Malaria Readmission		Total, n (%)	P value
	No, n (%)	Yes, n (%)	n = 739	
Knowledge on transmission of malaria				
Mosquito bite				
Do not Know	3 (0.6)	4 (2)	7 (0.9)	0.068
Know	538 (99.4)	194 (98)	732 (99.1)	
Presence of bad sewage				
Do not know	265 (49)	88 (44.4)	353 (47.8)	0.274
Know	276 (51)	110 (55.6)	386 (52.2)	
Playing in bad water				
Do not know	238 (44)	87 (43.9)	325 (44)	0.99
Know	303 (56)	111 (56.1)	414 (56)	
Drinking bad water				
Do not know	209 (38.6)	80 (40.4)	289 (39.1)	0.662
Know	332 (61.4)	118(59.6)	450 (60.9)	
Flies				
Do not know	151 (27.9)	48 (24.2)	199 (26.9)	0.319
Know	390 (72.1)	150 (75.8)	540 (73.1)	
Eating uncovered food				
Do not know	200 (37)	78 (39.4)	278 (37.6)	0.547
Know	341 (63)	120 (60.6)	461 (62.4)	
Sleeping with infected person				
Do not know	250 (46.2)	70 (35.4)	320 (43.3)	0.008
Know	291 (53.8)	128 (64.6)	419 (56.7)	
From the mother to the child by breast feeding				
Do not know	229 (42.3)	67 (33.8)	296 (40.1)	0.037
Know	312 (57.7)	131 (66.2)	443 (59.9)	
Protective measures used against malaria				
Use of mosquito net	533 (98.5)	194 (98)	727 (98.4)	0.606
Mosquito repellents	31 (5.7)	13 (6.6)	44 (6)	0.671
Close windows and doors	174 (32.2)	34 (17.2)	208 (28.1)	< 0.001
Burn cow dung/leaves	41 (7.6)	11 (5.6)	52 (7)	0.341
Wear long sleeve shirts	103 (19)	24 (12.1)	127 (17.2)	0.027
Clearing bushes	323 (59.7)	61 (30.8)	384 (52)	< 0.001
Draining stagnant water	303 (56)	60 (30.3)	363 (49.1)	< 0.001
Use Insecticide spray	123 (22.7)	19 (9.6)	142 (19.2)	< 0.001
Use Preventive medicine	57 (10.5)	37 (18.7)	94 (12.7)	0.003

### Prevalence of severe malaria readmission

The prevalence of readmission in children with severe malaria within six months of discharge was 26% (198/739), with a 95% confidence interval (CI) 23.6–30.1%. The median number of days to readmission was 97 (50.5–127) days.

### Clinical characteristics of the participants at previous and current admission

**Fig. 2:**
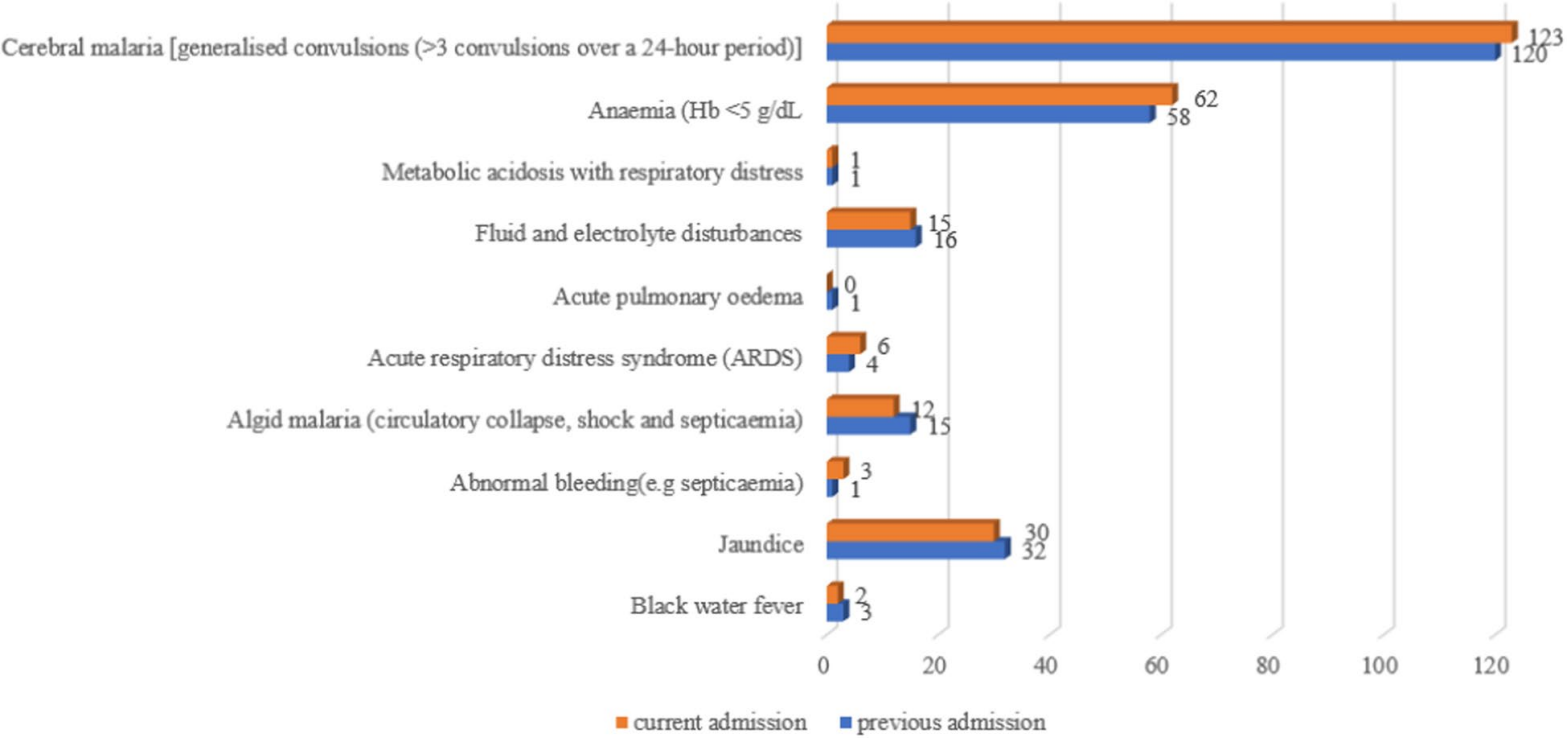
Clinical presentation at current and previous admissions of children under 5 years of age with severe malaria in Northern Uganda

Among the 198 children who were readmitted, 62% (123/198) had cerebral malaria, and 31.3% (62/198) had severe anaemia. Only three patients had black water fever (Fig. 2).

### Factors associated with severe malaria readmission among children under 5 years in in northern Uganda

The proportion of readmissions with severe malaria was 1.72 times greater among children with sickle cell disease [aPR 1.72; 95% CI (1.95–3.14)] than among those without sickle cell disease. The proportion of readmissions with severe malaria was 2.10 times greater among children who stayed in houses made of straw and grass thatch as wall material [aPR: 2.10; 95% CI (1.19–3.69)] than among those who stayed in houses with walls made of mud and poles and unburnt brinks. The proportion of readmissions with severe malaria was 2.01 times higher among those who seek care beyond 12 h when the child has a fever [aPR 2.01; 95% CI (1.23–3.29)] than those who sought care within 12 h (Table 3).

**Table 3:** Factors associated with severe malaria readmission among children under 5 years in Northern Uganda

Variable	cPR	95% CI	p value	aPR	95% CI	p value
Age of the child						
1 year	1.06	(0.75–1.50)	0.725	1.18	(0.92–1.54)	0.19
2 to 3 years	1			1		
4 years	1.02	(0.82–1.27)	0.849	1.15	(0.98–1.30)	0.092
Maternal education						
None/primary	1			1		
Secondary	0.86	(0.59–1.24)	0.415	1.60	(0.69–1.90)	0.604
Tertiary	0.82	(0.44–1.53)	0.525	1.47	(0.90–2.37)	0.120
Paternal education						
None/primary	1			1		
Secondary	0.78	(0.46–1.32)	0.358	0.98	(0.64–1.51)	0.928
Tertiary	0.55	(0.35- 0.88)	0.013	0.61	(0.31–1.20)	1.55
Wealth indices						
1	1.38	(0.85–2.22)	0.19	1.20	(0.82–1.67)	0.399
2	1			1		
3	0.74	(0.51–1.06)	0.102	0.87	(0.61–1.25)	0.461
4	0.57	(0.32–1.00)	0.052	0.81	(0.48–1.37)	0.431
5	0.73	(0.42–1.28)	0.268	0.95	(0.52–1.83)	0.955
Sickle cell disease						
No	1			1		
Yes	1.93	(1.01–3.69)	0.047	1.72	(1.95–3.14)	0.07
HIV						
No	1			1		
Yes	0.38	(0.10–1.41)	0.148	0.43	(0.13–1.46)	0.177
Wall material						
Straw and thatched	3.61	(2.11–6.15)	< 0.001	2.10	(1.19–3.69)	0.010
Mud & poles, unburnt brinks	1.32	(0.93–1.89)	0.124	1.08	(0.61–1.92)	0.79
Cement, burnt bricks, timber, stone	1			1		
Protective measures						
Use Insecticide spray						
No	1			1		
Yes	0.45	(0.27–0.74)	0.002	0.73	(0.26–1.41)	0. 263
Use of mosquito net						
No	1			1		
Yes	0.8	(0.28–2.30)	0.681	0.94	(0.44–1.99)	0. 879
Knowledge on malaria transmission						
Poor	1			1		
Good	1.21	(0.70–2.09)	0.496	1.28	(0.91–1.77)	0.155
Time to seeking care when the child has a fever						
Within 12 h	1					
Within 13–24 h	3.16	(1.80–5.61)	< 0.001	2.01	(1.23–3.29)	0.005
More than 24 h	1.90	(1.47–2.45)	0.027	1.65	(0.97–2.81)	0.064

cPR: crude prevalence ratio, aPR: adjusted prevalence ratio, p value: probability value

## Discussion

This study provides crucial insights into the prevalence and associated factors for readmission with severe malaria among children under 5 years of age in Northern Uganda within six months post-discharge. The readmission rate was high at 26.8% (198/739), reflecting the persistent threat of malaria in endemic regions [13]. Sickle cell anaemia, poor housing conditions (straw and thatch walls) and seeking care after 12 h when a child has a fever were significantly associated with an elevated risk of readmission, underscoring the multifaceted nature of the malaria burden.

The high readmission rate observed suggests that children remain at considerable risk of severe malaria episodes even after hospital discharge. This is likely exacerbated by the study's timing, which coincided with the peak malaria transmission season (March to May), when exposure to mosquito bites is heightened. The endemic nature of malaria in Northern Uganda [8] further highlights this risk, particularly in young children who have not yet developed sufficient immunity. These findings underscore the urgent need for post-discharge interventions, such as prophylactic anti-malarials, to reduce the risk of reinfection and recurrent hospitalization.

Children with sickle cell anaemia were more likely to be readmitted with severe malaria. All the sixty-seven children used mosquito bed nets. This finding is consistent with known vulnerabilities of children with sickle cell disease [14], who are physiologically predisposed to severe outcomes due to chronic anaemia and compromised immunity [15]. While similar associations have been reported in Kenya, this finding differs from that of a large trial in Uganda and Malawi, which reported a reduced risk of readmission for children with sickle cell disease. These conflicting results warrant further investigation into the interaction between sickle cell anaemia and malaria in different contexts.

Children living in houses made of straw and thatch were at significantly greater risk of readmission. The porous nature of these materials provides poor protection against mosquitoes, increasing exposure and vulnerability to reinfection [16]. Housing quality, as demonstrated in other studies from Tanzania and beyond, plays a critical role in malaria transmission dynamics and highlights the need for broader public health interventions targeting socioeconomic and environmental factors [17].

Seeking care after 12 h when a child has a fever increased the risk of readmission of children with severe malaria. Delay in seeking treatment for children with severe malaria is a key predictor for development of severe complications such as anaemia, kidney injury and cerebral malaria [18]. These complications further contribute to the recurrent hospitalization of children treated for severe malaria [19]. This finding is similar to another study in Eastern Uganda which associated development severe complications of severe malaria with delay in seeking treatment [20].

Despite these important findings, this study had some limitations. Comprehensive laboratory assessments, such as renal function tests and parasite density measurements, which may have provided a more nuanced understanding of the clinical manifestations were not performed. In addition, the data collection coincided with the malaria peak season, potentially inflating the readmission rate due to seasonal variations in transmission. Lastly, incomplete or missing data from some participants led to minor exclusions (2.8%), although this is unlikely to have introduced substantial bias.

Overall, this study underscores the high burden of severe malaria readmission in Northern Uganda and the need for targeted interventions, particularly among vulnerable populations, such as children with sickle cell anaemia and those living in inadequate housing conditions. Prophylactic strategies and improvements in living conditions in addition to use of insecticide-treated mosquito bed nets could significantly reduce the risk of recurrent malaria episodes and associated complications.

## Conclusion

The study concludes that severe malaria remains a significant challenge for children under 5 years of age in Northern Uganda, with a high readmission rate of 26.8% within six months post-discharge. Key risk factors for readmission include sickle cell anaemia and living in poor housing conditions (straw and thatch walls) and time to seeking care when the child has a fever all of which contribute to the elevated vulnerability of these children. This study highlights the importance of post-discharge interventions, such as prophylactic anti-malarials to prevent recurrent infections. There is also need to continue educating communities especially in endemic regions about the importance of fever as a key symptom of malaria. In addition, improvements in housing quality and broader public health strategies are essential for reducing the burden of malaria, particularly in endemic regions.

Further research is needed to understand the interaction between sickle cell anaemia and malaria, given the conflicting findings with those of previous studies. Despite some limitations, such as the timing of data collection and incomplete data, this study emphasizes the urgent need for targeted interventions to reduce the recurrence of severe malaria and its associated complications in high-risk populations.

## Data Availability

The datasets used and/or analyzed in this study are available from the first author upon reasonable request.

## Abbreviations

aPR: Adjusted prevalence ratio
CI: Confidence interval
cPR: Crude prevalence ratio
HIV: Human immunodeficiency virus
SD: Standard deviation
SSA: Sub-Saharan Africa

## References

[1] The VP. WHO World malaria report. Lancet Microbe. 2023;2024(5): e214. 10.1016/S2666-5247(24)00016-8 38309283

[2] Lalloo DG, Shingadia D, Bell DJ, Beeching NJ, Whitty CJM, Chiodini PL. UK malaria treatment guidelines 2016. J Infect. 2016;72:635–49. 26880088 10.1016/j.jinf.2016.02.001 PMC7132403

[3] WHO. Guidelines for the treatment of malaria. Geneva: World Health Organization. 2015. https://apps.who.int/iris/bitstream/handle/10665/162441/9789241549127_eng.pdf?sequence=1. 26020088

[4] Head MG, Goss S, Gelister Y, Alegana V, Brown RJ, Clarke SC, et al. Global funding trends for malaria research in sub-Saharan Africa: a systematic analysis. Lancet Glob Health. 2017;5:e772–81. 28668230 10.1016/S2214-109X(17)30245-0 PMC5567191

[5] Connon R, George EC, Olupot-Olupot P, Kiguli S, Chagaluka G, Alaroker F, et al. Incidence and predictors of hospital readmission in children presenting with severe anaemia in Uganda and Malawi: a secondary analysis of TRACT trial data. BMC Public Health. 2021;21:1480. 34325680 10.1186/s12889-021-11481-6 PMC8323322

[6] Kwambai TK, Mori AT, Nevitt S, van Eijk AM, Samuels AM, Robberstad B, et al. Post-discharge morbidity and mortality in children admitted with severe anaemia and other health conditions in malaria-endemic settings in Africa: a systematic review and meta-analysis. Lancet Child Adolesc Health. 2022;6:474–83. 35605629 10.1016/S2352-4642(22)00074-8 PMC10196725

[7] Chen F, Chen X, Gu P, Sang X, Wu R, Tian M, et al. The economic burden of malaria inpatients and its determinants during China’s elimination stage. Front Public Health. 2022;10: 994529. 36388376 10.3389/fpubh.2022.994529 PMC9651145

[8] Uganda National Malaria Control Division, Uganda Bureau of Statistics, and ICF. Uganda Malaria Indicator Survey 2018–19. Kampala, Uganda, and Rockville, Maryland, USA 2020.

[9] Staedke SG, Gonahasa S, Dorsey G, Kamya MR, Maiteki-Sebuguzi C, Lynd A, et al. Effect of long-lasting insecticidal nets with and without piperonyl butoxide on malaria indicators in Uganda (LLINEUP): a pragmatic, cluster-randomised trial embedded in a national LLIN distribution campaign. Lancet. 2020;395:1292–303. 32305094 10.1016/S0140-6736(20)30214-2 PMC7181182

[10] Tugume A, Muneza F, Oporia F, Kiconco A, Kihembo C, Kisakye AN, et al. Effects and factors associated with indoor residual spraying with Actellic 300 CS on malaria morbidity in Lira District, Northern Uganda. Malar J. 2019;18:44. 30791906 10.1186/s12936-019-2681-6 PMC6383239

[11] Ministry of Health Uganda. The Uganda malaria reduction strategic plan 2014–2020. Malaria control programme, Kampala, Uganda. 2015

[12] Namayanja C, Eregu EEI, Ongodia P, Okalebo CB, Okiror W, Okello F, et al. Unusual clinical spectra of childhood severe malaria during malaria epidemic in eastern Uganda: a prospective study. Malar J. 2023;22:169. 37259110 10.1186/s12936-023-04586-3 PMC10232340

[13] Zalwango MG, Bulage L, Zalwango JF, Migisha R, Agaba BB, Kadobera D, et al. Trends and distribution of severe malaria cases, Uganda, 2017–2021: analysis of health management information system data. Uganda National Institute of Public Health. Q Epidemiol Bull. 2023;8:2.

[14] Oppong M, Lamptey H, Kyei-Baafour E, Aculley B, Ofori EA, Tornyigah B, et al. Prevalence of sickle cell disorders and malaria infection in children aged 1–12 years in the Volta Region, Ghana: a community-based study. Malar J. 2020;19:426. 33228681 10.1186/s12936-020-03500-5 PMC7684914

[15] Booth C, Inusa B, Obaro SK. Infection in sickle cell disease: a review. Int J Infect Dis. 2010;14:e2–12. 19497774 10.1016/j.ijid.2009.03.010

[16] Ngadjeu CS, Doumbe-Belisse P, Talipouo A, Djamouko-Djonkam L, Awono-Ambene P, Kekeunou S, et al. Influence of house characteristics on mosquito distribution and malaria transmission in the city of Yaoundé, Cameroon. Malar J. 2020;19:53. 32000786 10.1186/s12936-020-3133-z PMC6993434

[17] Bofu RM, Santos EM, Msugupakulya BJ, Kahamba NF, Swilla JD, Njalambaha R, et al. The needs and opportunities for housing improvement for malaria control in southern Tanzania. Malar J. 2023;22:69. 36849883 10.1186/s12936-023-04499-1 PMC9972788

[18] Mousa A, Al-Taiar A, Anstey NM, Badaut C, Barber BE, Bassat Q, et al. The impact of delayed treatment of uncomplicated *P. falciparum* malaria on progression to severe malaria: a systematic review and a pooled multicentre individual-patient meta-analysis. PLoS Med. 2020;17:e1003359. 33075101 10.1371/journal.pmed.1003359 PMC7571702

[19] Opoka RO, Hamre KES, Brand N, Bangirana P, Idro R, John CC. High postdischarge morbidity in Ugandan children with severe malarial anemia or cerebral malaria. J Pediatric Infect Dis Soc. 2017;6:e41–8. 28339598 10.1093/jpids/piw060 PMC5907851

[20] Zalwango MG, Simbwa BN, Kabami Z, Kawungezi PC, Wanyana MW, Akunzirwe R, et al. Risk factors for death among children with severe malaria, Ivukula sub-county, Namutumba district, Eastern Uganda, September 2021–February 2022. Malar J. 2024;23:288. 39334376 10.1186/s12936-024-05111-w PMC11438375

